# Influence of pretesting and a near peer sharing real life experiences on CPR training outcomes in first year medical students: a non-randomized quasi-experimental study

**DOI:** 10.1186/s12909-022-03506-4

**Published:** 2022-06-06

**Authors:** Anne D Souza, Dhiren Punja, Sushma Prabhath, Akhilesh Kumar Pandey

**Affiliations:** 1 Department of Anatomy, Kasturba Medical College, Manipal, Manipal Academy of Higher Education, Manipal, India; 2 Department of Physiology, Kasturba Medical College, Manipal, Manipal Academy of Higher Education, Manipal, India; 3 Department of Community Medicine, Kasturba Medical College, Manipal, Manipal Academy of Higher Education, Manipal, India

**Keywords:** CPR training, Medical undergraduates, Peer interaction, Learning

## Abstract

**Background:**

Existing literature on cardiopulmonary resuscitation (CPR) training focuses on the knowledge and skill components while the attitudinal component is rarely addressed. There is a need to explore how peer interaction, learning atmosphere, and communication influence learning effectiveness during CPR training. Therefore, this study’s objective was to evaluate how a senior student (near peer) sharing their real-life experience of performing CPR would influence medical students’ learning and readiness to perform CPR.

**Methods:**

The present study involved 250 newly enrolled undergraduate medical students. The Solomon’s four-group study design was used to evaluate the influence of both pretesting and peer interaction. Students belonging to two groups initially completed a pre-training knowledge test (pretest) and a questionnaire on readiness to perform CPR. Students from all four groups then participated in instructor-led hands-on skills training, after which the two intervention groups interacted with their senior, who shared their life experiences of performing CPR. Finally, all four groups underwent skills evaluation, immediate and delayed post-tests, and completed a questionnaire to assess readiness to perform CPR. The students also provided their feedback on the experience of interacting with a peer during the training.

**Results:**

Post-test scores were significantly higher than pretest scores (Kruskal–Wallis test, *p* < 0.05). Scores were significantly higher in pretested intervention groups than in the non-pretested non-intervention group (*p* < 0.05). Delayed post-test scores were slightly but not significantly lower than the immediate post-test scores with no significant difference observed in the scores among the groups. The pretested groups showed more readiness to perform CPR and the pretested intervention group were least concerned about acquiring infection during CPR. Students in all groups were confident of performing chest compressions correctly, and found it inspiring and motivating listening to and discussing real-life experiences with a near peer.

**Conclusions:**

Hearing from peers about real-life CPR experience during CPR training sessions significantly impacted learning, enhanced student motivation to learn and may be an effective strategy to consider in routine CPR training. However, the positive effects of pretesting and peer interaction on knowledge were not sustained, highlighting a need for repeat training.

**Supplementary Information:**

The online version contains supplementary material available at 10.1186/s12909-022-03506-4.

## Background

Cardiopulmonary resuscitation (CPR) skill training is essential for healthcare providers, and a structured CPR training program exists in most medical schools worldwide, reflecting the importance of CPR skills in undergraduate students [1]. Early CPR training, when conducted with a holistic approach, has proven to improve confidence and skills among students of diverse healthcare disciplines [1–8]. Similarly, the National Medical Commission (NMC) has mandated a skills module as part of the foundation course for first-year medical undergraduates, emphasizing early CPR training to build and retain these essential skills [9].

Several active learning strategies such as traditional lectures, video self-instruction, serious games, and online modules are used to acquire CPR skills [10, 11]. Simulation is the most acceptable and feasible strategy, enhances the learning experience and makes learning engaging, resulting in better retention [7, 12]. Pretesting and pre-evaluation feedback have also been shown to influence CPR training learning outcomes [13–16].

Peer-led training is a cost-effective learning strategy which results in excellent student outcome and satisfaction [17–19]. Motivation to learn influences the student’s learning process [20]. These findings led us to explore how peer relationships, learning atmosphere, and communication influence the effectiveness of learning CPR.

Previous studies have shown that observing doctors communicating with their patients can enhance knowledge on effective doctor-patient communication strategies amongst medical students [21]. Vicarious experiences of this kind can help learners believe they can match the accomplishments of their peers, and impact on students is directly correlated with the extent to which they relate to the model being observed. Observational learning, vicarious experiences, and reflective peer coaching from near peers who are immediate seniors and therefore psychosocially and demographically similar but who are more competent and academically more advanced than the learner can instill self-efficacy in the learners [22, 23]. In some instances peer-led training has been shown to enhance performance and retention of skills [24].

New medical entrants are comfortable with and relate to near peers who serve as valuable role models and are known to influence the cognitive and psychomotor learning of the new learner in a positive manner, such as motivating them to study harder. Near peer interactions can help alleviate concerns of new medical students and enhance confidence in their skills to acquire knowledge. Near peer programs have been recommended for delivery of the curriculum throughout the medical undergraduate training program [25, 26]**.**

Therefore, we hypothesized that a near peer sharing their real-life experience of performing CPR on a patient will enhance learning in first year medical undergraduates, in terms of better knowledge evident in immediate and delayed post test scores, better skills assessment scores, enhanced self-efficacy in the individual components of CPR, enhanced readiness and reduced concerns performing CPR on a patient. We also aimed to study how pretesting influences knowledge and skills of CPR, readiness to perform CPR, self-efficacy and concerns regarding CPR in first-year medical undergraduates who did and did not undergo a near peer interaction.

This study also aimed to understand how students rate the influence of the near peer interaction on their learning.

## Materials and methods

The foundation course introduced by the NMC strongly highlighted building essential skills and professionalism at an early phase of the medical curriculum [9, 27]. To develop these skills and qualities, we designed a CPR skills training module for first year undergraduate medical students, incorporating skills with an attitudinal component, delivered in the first month of their foundation course.

### Conceptualization of the training module

We designed a knowledge questionnaire (See additional file 1) containing 15 multiple-choice questions (MCQs) each worth one mark. Then, to evaluate change in the student readiness, we designed another questionnaire titled ‘readiness to perform CPR’ using MCQs and visual analogue scale (VAS) questions (see Additional file 2). This questionnaire included eight VAS questions with scores ranging from 0 to 10. Two additional questions tested concerns regarding CPR and self -efficacy in individual components of CPR respectively. The sum of the VAS ranks for each student was a maximum of 80. A study by Beckers et al. was used as a guide to the type of questions [13]. Two faculty members with subject-related expertise and a statistician validated the questionnaires for relevance, clarity, and simplicity of each question.

We also developed a skill-evaluation checklist focused on the steps of adult CPR (see Additional file 3) and with a maximum score of 40. The checklist included four major scoring components: assessing response, chest compressions, breaths, and operating the automated external defibrillator (AED) and was based on previous research by Li et al. [15]. Two certified basic life support (BLS) instructors within the institution validated the checklist. We created links and barcodes for all the questionnaires using ‘Google forms.’ The students completed the questionnaires using their smartphones.

Cardiopulmonary resuscitation instructors were American Heart Association (AHA) certified pre- and para-clinical faculty trained in BLS. The instructors met three times prior to the intervention in the simulation lab to discuss the course design, method of content delivery, and checklist-based skills assessment. All instructors had a minimum of 5 years’ experience teaching AHA-certified programs to medical students.

One day prior to undergoing the skills training, students attended lecture halls for a 1-hour interactive lecture introducing the terminologies used in BLS and providing an overview of the BLS program. The instructor-led skills training was conducted at the Simulation Centre at a Medical College in South India using mannequins (Little Anne, Laerdal, USA) and mainly focused on one- and two-rescuer adult CPR individual skills. The training was in accordance with the 2015 American Heart Association guidelines for adult basic life support [28].

### Study design and setting

This non-randomized experimental study included 250 newly enrolled undergraduate medical students. The students gave their written informed consent before participating in the study during August 2019. The Institutional Ethics Committee (IEC) of Kasturba Medical College, Manipal (IEC 624/2019) approved the study.

Solomon’s four-group study design was used to evaluate the influence of pretesting and peer interaction [29]. We allocated the 250 students to groups A and B (125 in each group) for the interactive lecture session (Session 1). In addition, students in group ‘A’ completed a pretest and a questionnaire on readiness to perform CPR and concerns regarding CPR immediately before the lecture.

After the lecture, the students were divided into four small groups A1, A2, B1, and B2 (62–63 students in each group) for skills training. The skills training session was conducted over four consecutive days. Each group underwent the training on a different day but with the same set of instructors which helped maintain constancy in the content objectives, depth of coverage, and pedagogical style. The instructor-to-student ratio was 1:10. During the 4-hour training session the instructors demonstrated individual skill components, and each student had opportunities to practice these skills on the CPR mannequins (Session 2).

Students in groups A2 and B1 interacted with their senior, a third-year undergraduate student who shared her life experiences of performing CPR on cardiac arrest patients. She recounted two such incidents which arose while traveling, one in an airplane and the other on a train. During the initial 15–20 minutes of the training, she spoke about her initial apprehensions to perform CPR. She explained how recollecting her earlier CPR training in the institute gave her a sense of preparedness and motivated her to approach the victim and perform CPR, and how her initiative to begin CPR on the victim motivated others around her to help. She also spoke about the sense of accomplishment she gained from the fact that her efforts to save the patient were successful in one of the two situations that she had experienced, and how the patient’s family members acknowledged her life-saving role. The students then had the opportunity to interact with her in a question-answer session. In contrast, students in groups A1 and B2 underwent the skills training without peer interaction.

All four groups took a knowledge test immediately after training (the post-test) followed by a scenario-based skills evaluation of one and two-rescuer adult CPR. They also completed the questionnaire on readiness to perform CPR, concerns regarding CPR, and self-efficacy in individual CPR skills. Feedback on the near peer interaction was gathered from the relevant groups (A2 and B1) using open-ended questions (Additional file 2).

**Fig. 1:**
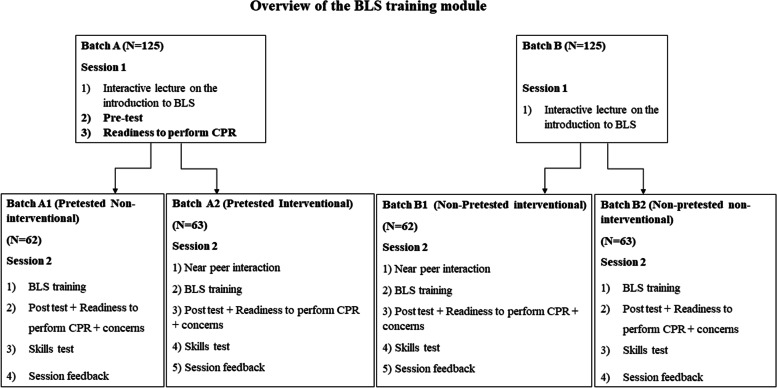
A schematic flow diagram providing an overview of the BLS training module

A delayed post-test was conducted 4 weeks after the skills training to test long-term knowledge retention. Figure 1 provides an overview of the training module and the group allocation.

### Data analysis

Since data deviated from a normal distribution, non-parametric tests were used in statistical analyses. To evaluate how the pretesting and near peer interaction influenced learning and readiness, we compared the immediate and delayed post-test scores, skill scores, and readiness to perform CPR between the four groups using Kruskal–Wallis with post-hoc analysis using Mann-Whitney U test for pair-wise comparison.

Students’ concerns about and self-efficacy in performing CPR were analyzed using a Chi square test. Analyses were conducted using the statistical software ‘EZR’ (Easy R), a graphic user interface for R software (The R Foundation for Statistical Computing, Vienna, Austria) [30].. The data were expressed as mean and standard deviation with 95% confidence interval (CI). A *p*-value < 0.05 was considered as statistically significant.

We also performed a thematic analysis of feedback on the peer interaction. The researchers read, re-read, and manually coded the participant responses using an inductive and semantic approach [31]. The codes were studied to identify patterns and themes. The datasets generated and analyzed during the current study are available in the Mendeley Data repository, [https://data.mendeley.com/datasets/r6fskjgxrj/2] [32].

## Results

Two hundred and fifty first-year medical undergraduate students participated in the study (126 males and 124 females; age range 18–23 years). Two hundred and forty-two (96.8%) students were of Indian origin, and the remaining eight (3.2%) were foreign citizens. Out of 109 respondents to the questionnaire that was given before the training, 80 (73.3%) students had heard of BLS before attending medical school. Of these, 34 (31.2%) had obtained information from the internet and media sources, 23 (21.1%) from their school, and 13 (11.9%) students from their parents who were doctors by profession. Sixty-five students (59.6%) were aware of the AED device. Prior BLS training had been undertaken by six (5.5%) international students and none of the Indian students. None of the students had experience performing CPR on a real patient. However, seven (6.4%) students had witnessed someone else performing CPR on an individual in emergencies.

Of the 125 students in group A, who attended the interactive lecture, 107 (85.6%) completed the pretest and 109 (88%) completed the questionnaire on readiness to perform CPR. Of the 250 students who participated in the skills training session, 243 (97.2%) answered the immediate post-test, and 237 (94.8%) completed the questionnaire on readiness to perform CPR. However, only 219 (87.6%) completed the delayed post-test.

### Influence of near peer interaction and pretesting on knowledge test scores

Among the whole cohort, knowledge scores were 5.16 ± 1.69 (range 2–12; *N* = 107) at pretest, 12.17 ± 1.54 (range 8–15; *N* = 243) at post-test, and 10.34 ± 2.10 (range 4–15; *N* = 237) at delayed post-test.

Post-test scores were highest in the pretested intervention group (A2; 12.82 ± 1.22) and lowest in the non-pretested, non-intervention group (B2; 11.38 ± 1.31). Post-hoc analysis with Mann-Whitney U test showed that post-test scores were significantly higher in the pretested non-intervention, pretested intervention and non-pretested intervention groups, (A1, A2, and B1) than in the non-pretested non-intervention group (B2). However, the delayed post-test scores were not significantly different between the groups. Test scores and comparisons between the groups are shown in Table 1.

**Table 1:** Comparison of pre-test, immediate post-test and the delayed post-test scores between intervention and non-intervention groups

Test	Intervention groups			Non-intervention groups			H value (Kruskal- Wallis test)	*p* Value (Kruskal- Wallis test)	Mean difference and 95% Confidence interval
	Pre-tested (A2)	Non pre-tested (B1)	Combined scores	Pre-tested (A1)	Non pre-tested (B2)	Combined scores			
Pre-test	5.11 ± 1.53	NA	NA	5.23 ± 1.88	NA	NA	NA	0.82	0.12 (−0.53 to 0.77)
Immediate post-test	12.82 ± 1.22°	12.23 ± 1.68¤	12.53 ± 2.21	12.22 ± 1.58*	11.38 ± 1.31*°¤	11.80 ± 2.28	28.293	< 0.001	−0.77 (−1.15 to − 0.39)
Delayed post-test	11.03 ± 1.86	9.92 ± 2.08	10.20 ± 2.07	10.51 ± 2.14	9.8 ± 2.14	10.50 ± 2.11	7.613	0.05	0.29 (−0.26 to 0.85)

*NA* Not applicable

*p = 0.02, ° *p* < 0.001, ¤ *p* = 0.002 as calculated using the Mann-Whitney U test. *P* value less than 0.05 was considered statistically significant

### Influence of near peer interaction and pretesting on skill test scores

All groups performed similarly well in skills evaluation, with a statistically similar mean score of 35.47 (out of 40) in the intervention groups and 35.26 in the non-intervention groups (mean difference − 0.21, 95% CI − 1.28 to 0.85).

### Influence of near peer interaction and pretesting on readiness to perform CPR

The whole cohort mean rank of VAS for the readiness to perform CPR before the session was 68.95 ± 8.46 (range 41–80; *N* = 109) and after the session was 71.57 ± 7.13 (range 43–80; *N* = 237).

A detailed analysis of responses after BLS revealed a significant difference between the four groups (Kruskal–Wallis test, H = 11.60, *p* = 0.008). Post-hoc analysis using the Mann-Whitney U test showed that the sum rank in the pretested non-intervention group (A1) was significantly higher than in the non-pretested groups (B1 and B2). Students in the pretested non-intervention group (A1) were significantly more ready than the non-pretested non-intervention group (B2) to attend CPR training at their own expense. Students in the pretested non-intervention group (A1) were more ready than the pretested intervention group (A2) to look for additional material on CPR. Both intervention and non-intervention groups were equally ready to perform CPR on a patient who is a stranger, but pretested groups showed more readiness (A1 and A2). Students in all four groups were equally ready to perform CPR on a family member when needed. The detailed ranks of responses on readiness are shown in Table 2.

**Table 2:** Mean and standard deviations of ranks of readiness questions after training with statistical comparison between the groups

Statement	Intervention groups		Non-intervention groups		H value and ***p*** value (Kruskal- Wallis test)	***P*** value for pairs with significant difference (Mann-Whitney U test)
	Pretested (A2)	Non-pretested (B1)	Pretested (A1)	Non-pretested (B2)		
How important do you think is it for all people to know how to perform CPR?	9.57 ± 0.87	9.76 ± 0.65	9.4 ± 1.12	9.64 ± 0.81	H = 4.03 *P* = 0.257	NA
How likely are you to attend a certified BLS training session at your own expense?	7.69 ± 2.15	7.62 ± 2.54	8.56 ± 2.12*	7.31 ± 2.47*	H = 11.75 *P* = 0.008	Between A1 and B2 (*p* = 0.009)
How likely are you to encourage other people to learn CPR?	9.11 ± 1.64	9.40 ± 1.05	9.25 ± 1.21	9.08 ± 1.45	H = 0.65 *P* = 0.883	NA
How likely are you to read or look up for additional material regarding CPR, after this session?	7.34 ± 2.46*	7.66 ± 2.20	8.45 ± 2.06*	8.03 ± 2.09	H = 10.39 *P* = 0.015	Between A1 and A2 (*p* = 0.026)
How likely are you to volunteer to train others to do CPR?	8.54 ± 1.76	8.71 ± 1.45	9.04 ± 1.32	8.75 ± 1.47	H = 2.15 *P* = 0.540	NA
If the victim is, a stranger how likely is it that you will perform CPR?	9.44 ± 0.87	9.03 ± 1.40	9.46 ± 0.66*	8.94 ± 1.12*	H = 11.71 *P* = 0.008	Between A1 and B2 (*p* = 0.028)
If the victim of a cardiorespiratory arrest, is your family member, how likely is it that you will perform CPR	9.77 ± 0.72	9.61 ± 0.94	9.74 ± 0.65	9.42 ± 1.22	H = 2.9 *P* = 0.405	NA
If you have adequate knowledge and skills, how likely is it that you will perform CPR on your own to a victim in need.	9.61 ± 0.76	9.22 ± 1.21	9.66 ± 0.66	9.22 ± 1.03	H = 2.2 *P* = 0.100	NA
Total Score	71.26 ± 7.58	70.87 ± 6.46*	73.49 ± 7.04*°	70.60 ± 7.26°	H = 11.60 *P* = 0.008	Between A1 and B1 (*p* = 0.025) And between A1 and B2 (*p* = 0.018)

*NA* Not applicable

*° Significance between the groups as calculated by the post-hoc analysis using Mann-Whitney U test. *P* value less than 0.05 was considered statistically significant

### Influence of near peer interaction and pretesting on concerns and self-efficacy in performing CPR

**Fig. 2:**
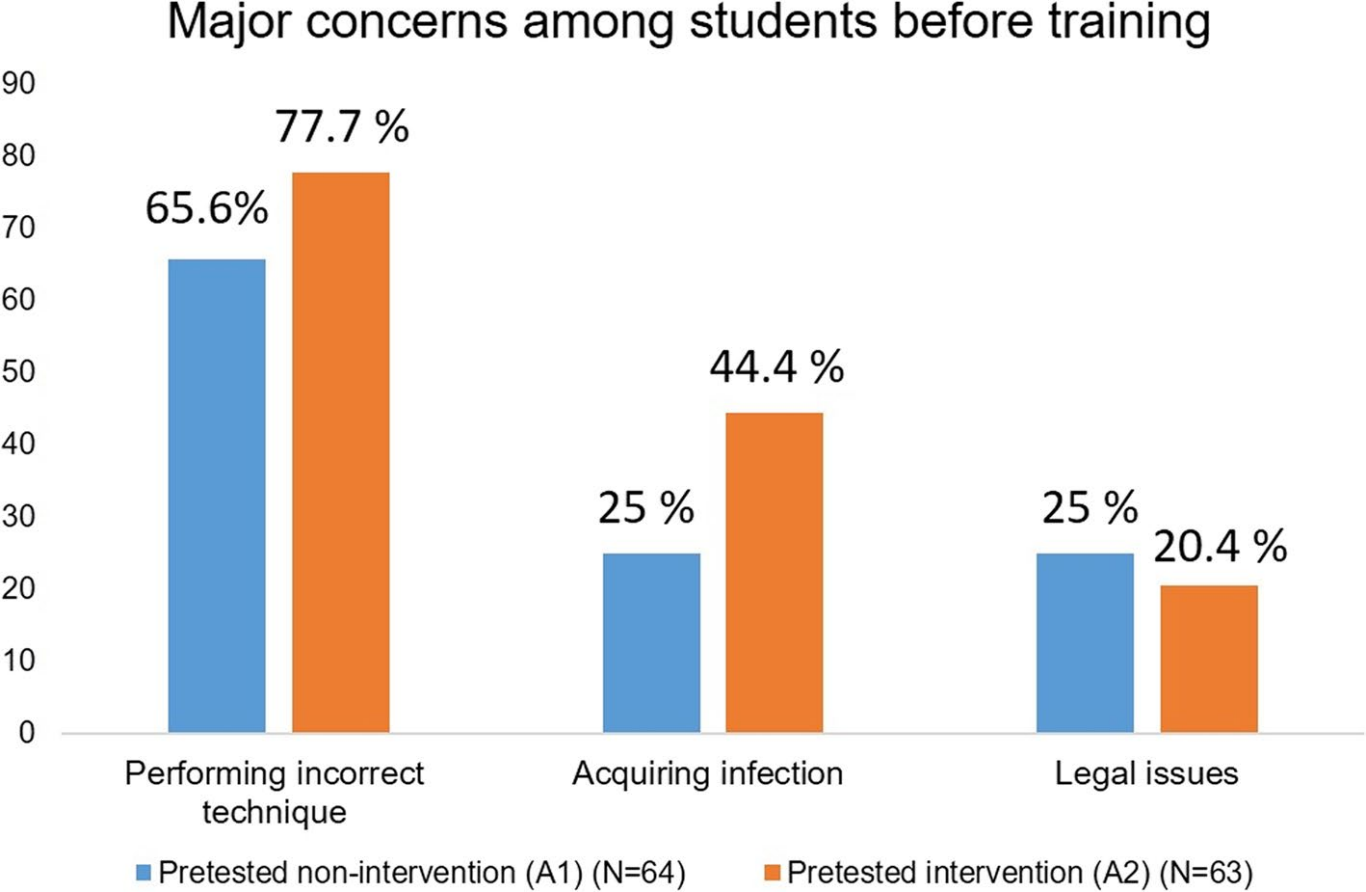
Frequency distribution of the significant concerns about CPR among the pretested groups before training

**Fig. 3:**
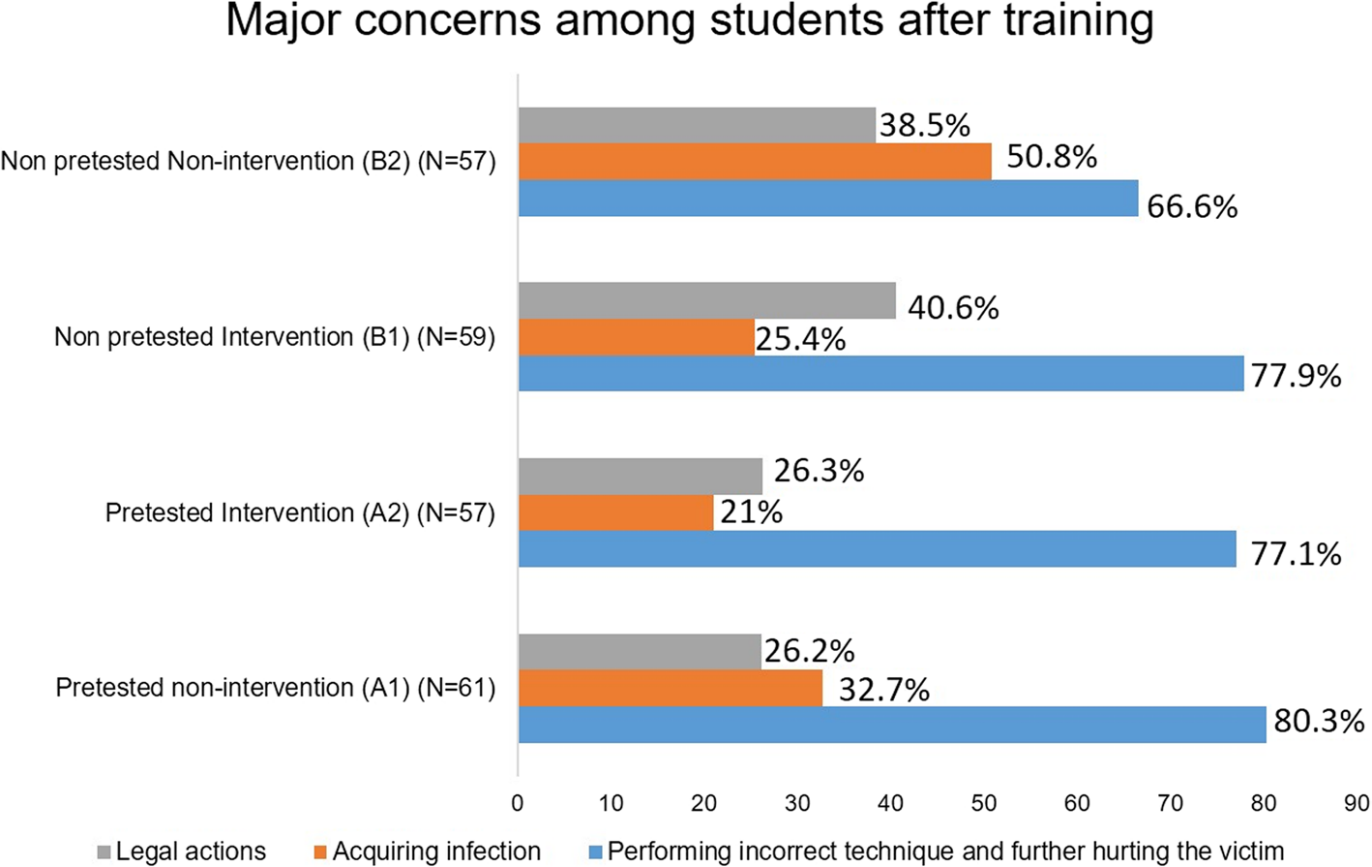
Frequency distribution of the significant concerns about performing CPR among all four groups after training

Figure 2 shows the frequency of students’ concerns about performing CPR gathered before training. Students in the intervention group (A2) had more concern about acquiring infection (44.4%) than those in the non-intervention group (A1) (25%). Frequency of concerns after training are shown in Fig. 3. Fear of performing the CPR technique incorrectly remained a significant concern in each group even after training (Kruskal-Wallis test, H = 3.451, *p* = 0.32). However, concern about acquiring infection was reduced in intervention groups (Kruskal -Wallis test, H = 13.476, *p* = 0.003).

**Fig. 4:**
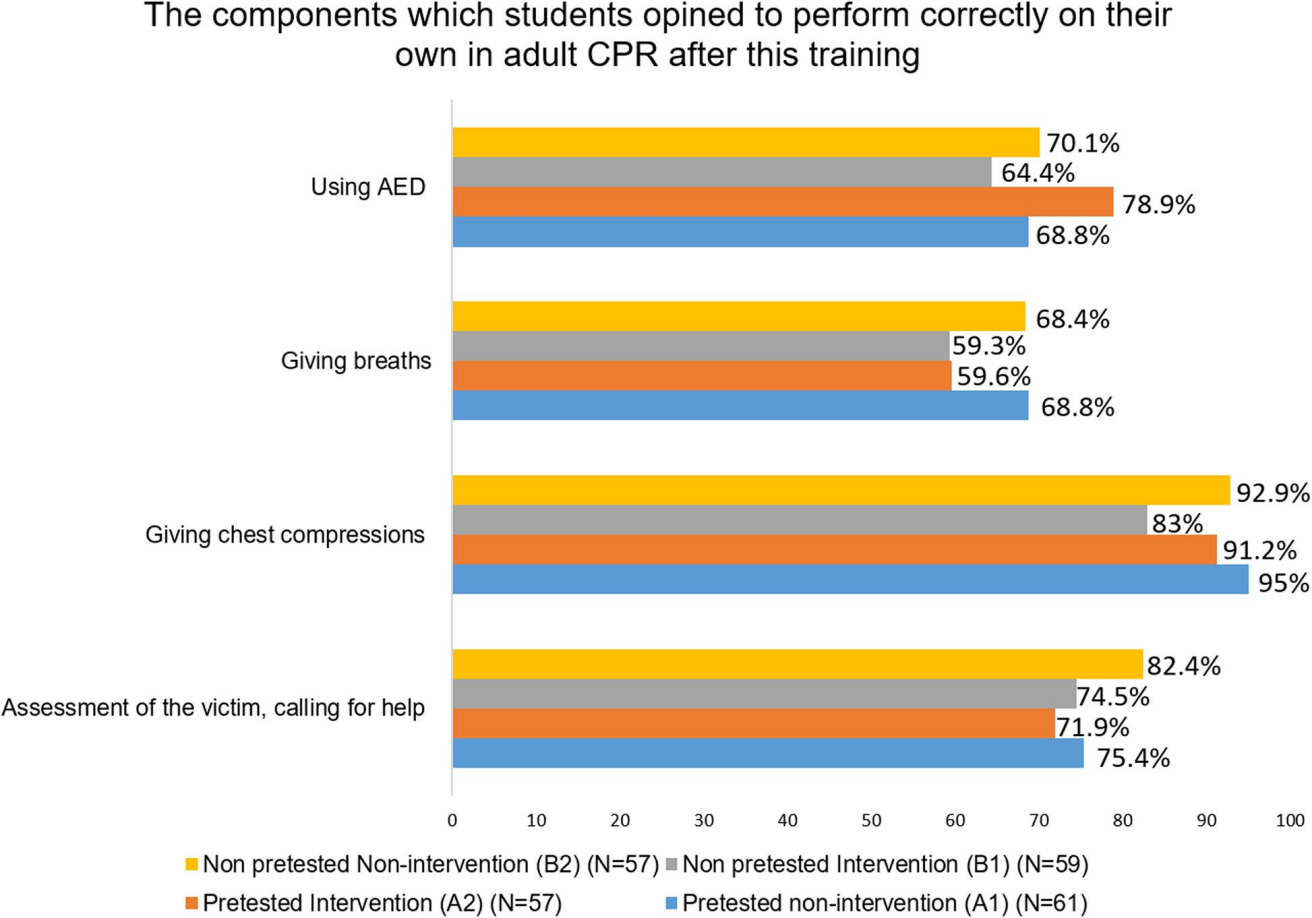
Frequency distribution of the post-training perceived likelihood of correctly performing the different steps of CPR (Self efficacy)

Figure 4 shows the distribution of students’ post-training self-efficacy in correctly performing steps of CPR. Although the intervention groups showed slightly lower self-efficacy, this difference was not statistically significant.

### Thematic analysis of feedback on near peer interaction

Most students indicated that hearing from the medical student about her experience of CPR motivated them to learn CPR (VAS rank 9.44 ± 1.48) and influenced their attitude towards CPR (9.35 ± 1.55). In addition, the students found it inspiring and motivating to interact with their peer and discuss real-life experiences.

The following five themes on the near peer interaction were identified: ‘Learning motivation/Inspiration’ (12 students); ‘Helping/Saving lives’ (18 students); ‘Change in attitude’ (six students); ‘Awareness’ (15 students); and ‘Knowledge gain’ (three students). For example, one student responded with “*It was truly inspiring, and after listening to it, I would highly recommend everyone to learn CPR*” (male student). Another male student stated, “*It proved that how even a student can save a life or at least try in dire situations.*” In addition, the session had an impact on students’ readiness towards performing CPR. “*It helped me change the attitude towards CPR*” (female student). Finally, the students found it inspiring to learn CPR. “*It inspired us to learn CPR, and her experience made us realize the real importance of CPR*” (male student).

Discussing the real-life scenario made students realize that emergencies can happen in any situation. “*Made me realize that emergencies do happen in real life, and it can happen around me anytime anywhere*” (female student). The students also became aware of the importance of life-saving CPR training. “*Most people do not have the correct CPR training, which affects a person’s life in an emergency*” (male student). In addition, the training enhanced their knowledge regarding BLS. “*It helped me enhance my knowledge and help in further medical supervision*” (female student). A table showing details of coding and themes is provided in Additional file 4.

## Discussion

Although medical students worldwide are trained in CPR skills, they have been found to lack knowledge about cardiac arrest [33]. Early CPR training for schoolchildren and in community education has improved bystander CPR and increased the outside-hospital cardiac arrest survival rate [34, 35]. Considering the wide range of CPR learning strategies currently used, incorporating innovative practices would make learning enjoyable and enhance the learning outcome. In the Solomon’s four group design used in this study, two of the groups were pretested. One pretested and one non-pretested group underwent the peer intervention, and all four groups were post-tested. This study design allowed us to study the main effects of the intervention and the pretesting and to study the interaction of pretesting and the intervention [36].

The results of the study revealed that having either the pretest or the near peer interaction enhanced knowledge gain and short-term knowledge retention, and that undergoing both the pretest and the interaction is more advantageous than having neither. However there seems to be no added benefit from pretesting together with peer interaction.

A survey conducted by Phadraig et al. revealed low levels of CPR knowledge and skills but high confidence among health sciences students, and indicated a need for timely training to develop CPR competencies [37]. In contrast, a study by Freund et al. found low confidence levels among medical students in performing CPR [5]. Our BLS training had a short-term positive impact on readiness to perform CPR, in agreement with a previous study [38]. While peer interaction did not influence this outcome, pretesting did influence this. Note that the pretested groups also underwent a pre-BLS readiness assessment. Previous studies do not show clearly whether pre-training attitude assessment has an influence on post-training attitude. The information on readiness conveyed in the pre-training assessment may evoke the learner’s curiosity and stimulate them to consider the implications of this information causing a priming effect that may induce attitude change [39, 40]. In the present study, the peer intervention did not influence self-efficacy in the individual components of CPR. Studies have shown that task specific self-efficacies may be influenced by generalized self-efficacy of the population under study [41].

Even though students were educated during training about CPR’s benefit-risk ratio, the possibility of harming the patient by performing the technique incorrectly remained the primary concern, consistent with previous studies [38]. In our study, students had more confidence in chest compression skills than giving breaths (Fig. 4). Despite being given information on the low incidence of acquiring infection with CPR and precautions to minimize incidence, the pretested non-intervention group showed more concern about infection after than before training, consistent with previous findings [38]. However, concern about acquiring infection was reduced in the pretested intervention group after training, which may be attributed to the influence of peer interaction.

Our finding that pretesting positively influenced student learning has been demonstrated in previous research [13, 15], De Ruijter found poor retention after 1 and 2 years of BLS training and advised shorter intervals between training sessions and early exposure to real-life emergencies for better skill retention [42]. The present study showed good CPR knowledge retention a month after training. However, the positive influences of pretesting and peer intervention on CPR knowledge were not preserved. We did not evaluate the retention of skills, which is one of the limitations of the current work.

Tu and Chu revealed a positive impact of peer relationships in learning motivation [20]. In the present study, listening to the near peer’s real-life experience had a positive impact on short-term retention of CPR knowledge. Students indicated that they found the account relatable as it came from someone in a role they would occupy in 2 years’ time. The fact that they underwent the same CPR training as the near peer gave them more confidence in their skills. Studies have shown that confidence in one’s skills predicts the likelihood of successfully applying these skills [22]. Hearing about personal accomplishment from a more advanced and competent yet socially and cognitively congruent peer can make students believe they can achieve the same success through perseverance, leading to enhanced learning motivation [22].

In the present study, students realized that emergencies may happen at any time, anywhere and CPR training failures can impact a person’s life in such situations. Providing emotional stimuli with implied social importance can impact memory and attention functions and potentially amplify the learning process [22]. Listening to an account of a person who has been saved by CPR can induce empathy and relief emotions, which are vital for social attachment [43]. Vicarious experiences of performing CPR can give students a secondary appraisal of their lack of skills in dealing with such situations, leading to heightened emotional activation and motivating them to focus on developing the necessary skills [22]. Discussing the near peer’s real-life scenarios made students realize the importance of having the power to save a life and motivated them to learn CPR better and to view it less casually. Two recounted scenarios in which the near peer was able or unable to save a life made the account feel more realistic to students, probably because it underscored the reality that not every life can be saved despite the best efforts.

Vicarious experiences are useful for the development of skills in which real experiences may be both scarce and diverse and where the need for reliable performance is high such as performing CPR. Interactions where near peers share their own experience of handling situations allows for exchange of ideas and enables collective learning. The thought processes, decisions, or intuitive feelings that a near peer shares may help transfer tacit prospective knowledge to the listener. This helps the learner navigate the uncertainty and complexity associated with certain tasks encountered during training and clinical practice [44]. Successful and effective CPR training results from several factors such as effective simulation, pretesting, peer interaction, smaller training groups, immediate feedback and periodic, repetitive training for better retention of knowledge and skills [10, 13, 40, 45]. The current study explored an innovative strategy in which a peer shared their real-life experience of performing CPR, we studied its influence on learning, and analyzed the influence of pretesting. Rigorous studies will be needed in the future to explore the impact of such an intervention on the retention of BLS knowledge and skills.

### Limitations

Several instructors carried out the skills assessments, and subjective differences may have influenced the skill scores. However, subjectivity was reduced by using a checklist for assessment. The delayed post-test assessed only knowledge and did not re-evaluate skills.

## Conclusion

The present study found that both pretesting and incorporation of near peer interaction has significant short-term positive effects on CPR knowledge. Although a change in readiness was observed after training, it did not significantly influence the attainment of skills. Students found the peer interaction inspiring and motivating. However, the positive effects of the pretesting and peer interaction on knowledge were not sustained, highlighting a need for repeat training.

## Supplementary Information

**Additional file 1.**

**Additional file 2.**

**Additional file 3.**

**Additional file 4.**

## Data Availability

The datasets generated and analysed during the current study are available in the Mendeley Data repository, [https://data.mendeley.com/datasets/r6fskjgxrj/2].

## Abbreviations

CPR: Cardiopulmonary resuscitation
NMC: National Medical Commission
MCQs: Multiple-choice questions
VAS: Visual analogue scale
AED: Automated external defibrillator
BLS: Basic life support
AHA: American Heart Association
IEC: Institutional ethics committee
