# Supplementary material for: Influence of pretesting and a near peer sharing real life experiences on CPR training outcomes in first year medical students: a non-randomized quasi-experimental study
Source: BMC Med Educ. 2022 Jun 6;22:434. doi: 10.1186/s12909-022-03506-4 (PMC9172151; doi:10.1186/s12909-022-03506-4)
Supplement: Supplementary file 1 — Additional file 1. [file 12909_2022_3506_MOESM1_ESM.docx]

**Knowledge pre-post-test questionnaire**

**BLS QUESTIONNAIRE- PRETEST**

Name: _________________________ Age: ______ Roll No: _____________Gender: ___

Place of origin_______________________________

- Tick one best response
- Kindly note the abbreviations

BLS- Basic life

CPR- Cardiopulmonary resuscitation

EMS- Emergency medical services

AED – Automated external defibrillator

1. When would you initiate cardiopulmonary resuscitation in an adult? (unresponsive means the person does not respond when you call out loud or tap the victim in order to wake up the person)

1. When the person is unresponsive with no breathing and no pulse*
2. When the person is unresponsive, breathing and pulse is present but weak
3. Person is responsive and has chest pain and is breathlessness
4. In all of the above cases
5. What is the emergency medical services number in India?
6. 100
7. 102
8. 104
9. 108*
10. If you see a person collapse suddenly, what would you do first?
11. Assess for responsiveness*
12. Check pulse
13. Call for help
14. Don’t know
15. Which of the following is the correct sequence to follow when you begin CPR? (in the options below, Airway refers to opening up the airways

Breathing refers to giving breaths to the victim and Circulation refers to giving chest compression to the victim)

1. Airway-breathing-circulation (ABC)
2. Breathing-airway-circulation (BAC)
3. Circulation-airway-breathing (CAB) *
4. Breathing-circulation-airway (BCA)

5) The best location for checking the pulse in an adult victim who you suspect has cardiac arrest is

1. Carotid pulse in the neck *
2. Radial pulse at the wrist
3. Brachial pulse at the elbow
4. Femoral pulse in the thigh
5. Which of the following is the correct ratio of chest compressions to ventilation for CPR in an adult victim?
6. 20:2
7. 30:2*
8. 30:1
9. 15:2
10. Which of the following is the correct site in front of the victim’s chest for giving chest compressions in CPR?
11. Right half of the chest
12. Left half of the chest
13. In the midline, upper half of the chest
14. In the midline, lower half of the chest *
15. What is the preferred method to open the airway of a victim of cardiac arrest with no suspected cervical spine injury?
16. Head tilt only
17. Head tilt & chin lift*
18. Jaw thrust
19. Chin lift only
20. What is the correct rate of chest compressions (number of compressions/minute) to be given to an adult victim of cardiorespiratory arrest?
21. 60 -80
22. 80 -100
23. 100-120 *
24. 120-140
25. The correct chest compression depth for adult victims should be at least
26. 2 cm
27. 5 cm *
28. 7 cm
29. 10 cm
30. What would you do when a person is unresponsive, is not breathing but has a weak pulse?
31. Give both chest compressions and breaths in the ratio of 30 :2
32. Give rescue breaths with no chest compressions and keep assessing pulse at regular intervals *
33. Give both chest compressions and breaths in the ratio of 15:2
34. Do not give breaths immediately, continue to check pulse and give CPR if pulse disappears
35. A person suddenly collapses at an airport and is attended by a doctor. The doctor confirms that the patient is in cardiac arrest and asks a bystander to call the ambulance and get an AED, while the doctor immediately begins to perform CPR. The AED is available at the airport and the bystander fetches it. When should the doctor use the AED?
36. As soon as it becomes available for use*
37. After 2 minutes of CPR
38. After the arrival of EMS personnel
39. Doctor need not use the AED
40. Which of the following is the correct sequence for operating the AED?
41. Switch on the AED device---attach electrode pads----analyze heart rhythm-----clear the victim and deliver shock*
42. Attach electrode pads---- Switch on the AED device ---analyze heart rhythm---clear the victim and deliver shock
43. Switch on the AED device –---attach electrode pads-----clear the victim and deliver shock-----analyze heart rhythm
44. Attach electrode pads------ Switch on the AED device –--clear the victim and deliver shock-----analyze heart rhythm
45. What is the principle behind giving good chest compression in CPR?
46. It helps push blood out of the heart and ensures circulation to vital organs *
47. It activates an arrested heart so that it can start beating once again on its own
48. It help push air in and out of the lungs
49. All of the above
50. What is the idea behind shocking the victim of cardiac arrest using an AED?
51. Activates the central nervous system and ensures the victim becomes conscious
52. Converts an abnormal heart rhythm into a normal heart rhythm *
53. Activates the nerves that supply the heart so that the heart starts beating again
54. Revives the muscles of respiration so that the person begins to breathe again

*Correct answer to each question
