# Supplementary material for: Influence of pretesting and a near peer sharing real life experiences on CPR training outcomes in first year medical students: a non-randomized quasi-experimental study
Source: BMC Med Educ. 2022 Jun 6;22:434. doi: 10.1186/s12909-022-03506-4 (PMC9172151; doi:10.1186/s12909-022-03506-4)
Supplement: Supplementary file 2 — Additional file 2. [file 12909_2022_3506_MOESM2_ESM.docx]

**VAS scoring sheet**

**BLS readiness questionnaire (Before the session)**

**INSTRUCTIONS & CONSENT FORM**

Please read this form carefully. If you decide to volunteer to take part, you must write your name in the informed consent document below. This will be retained for documentation purpose.

This is a questionnaire constructed to obtain the attitude of the students during the course of BLS training. This questionnaire will be given to all the first year MBBS (Bachelor of Medicine and Bachelor of Surgery) students at different points during the BLS training.

Fifteen questions would take approximately 5-10 minutes to answer.

The response obtained shall be handled confidentially. No student shall be identified based on the name or the role number.

Filling the questionnaire is voluntary. You may decline participation without giving any reason. Your participation will not bestow upon you any competitive academic or occupational advantage over other students or staff who do not volunteer, and we will not impose any academic or occupational penalty on those students or staff who do not volunteer.

If you agree to complete the questionnaire, please type your name in the box given below.

BLS- Basic life support

CPR- Cardiopulmonary resuscitation

EMS- Emergency medical services

AED – Automated external defibrillator

1. Age: __________
2. Gender: ____________
3. Roll No____________
4. Have you heard of BLS or CPR before coming to Manipal **Yes /No**

If yes from where /from whom?

1. Have you ever seen someone perform CPR on a real victim **Yes/No**
2. Have you ever performed CPR on a real victim **Yes /No**
3. Have you heard of or seen a device called the AED before coming to Manipal

**Yes /No**

If yes where /from whom?

1. Have you ever undergone training in BLS/CPR before? **Yes/No**

If Yes**,** - When? __________________________

- Where? ________________________

- Who gave the training? ____________________________

- How many times have you undergone training? _________

**RATING (VISUAL ANALOGUE SCALE) FROM 0-10**

1. If you have adequate knowledge and skills, how likely is it that you will perform CPR on your own to a victim in need.

Not likely very likely

1. If the victim of a cardiorespiratory arrest, is your family member, how likely is it that you will perform CPR.

Not likely very likely

1. If the victim is a stranger how likely is it that you will perform CPR?

Not likely very likely

1. What will you be most concerned about while performing CPR on a stranger (you can tick more than one option )
2. Performing incorrect technique and further hurting the victim
3. Acquiring infection
4. Legal actions
5. Any other (specify)
6. How important do you think is it for all people to know how to perform CPR?

Not important very important

1. How likely are you to read or look up for additional material regarding CPR, after this session?

Not likely very likely

1. How likely are you to encourage other people to learn CPR?

Not likely very likely

8) When do you think is the ideal time for you to learn CPR

a) Now is the correct time

b) Should have got the opportunity to learn earlier

c) Should be learnt later

9) How likely are you to attend a certified BLS training session at your own expense?

Not likely Very likely

10) How likely are you to volunteer to train others to do CPR?

Not likely Very likely

**BLS readiness questionnaire (After the session)**

**INSTRUCTIONS & CONSENT FORM**

Please read this form carefully. If you decide to volunteer to take part, you must write your name in the informed consent document below. This will be retained for documentation purpose.

This is a questionnaire constructed to obtain the attitude of the students during the course of BLS training. This questionnaire will be given to all the first year MBBS students at different points during the BLS training.

Fifteen questions would take approximately 5-10 minutes to answer.

The response obtained shall be handled confidentially. No student shall be identified based on the name or the role number.

Filling the questionnaire is voluntary. You may decline participation without giving any reason. Your participation will not bestow upon you any competitive academic or occupational advantage over other students or staff who do not volunteer, and we will not impose any academic or occupational penalty on those students or staff who do not volunteer.

If you agree to complete the questionnaire, please type your name in the box given below.

BLS- Basic life support

CPR- Cardiopulmonary resuscitation

EMS- Emergency medical services

AED – Automated external defibrillator

Age: __________

Gender: ____________

Roll No____________

1. If you have adequate knowledge and skills, how likely is it that you will perform CPR on your own to a victim in need.

Not likely (0) very likely (10)

1. If the victim of a cardiorespiratory arrest, is your family member, how likely is it that you will perform CPR.

Not likely very likely

1. If the victim is, a stranger how likely is it that you will perform CPR?

Not likely very likely

1. What will you be most concerned about while performing CPR on a stranger (you can tick one or more options )
2. Performing incorrect technique and further hurting the victim
3. Acquiring infection
4. Legal actions
5. Any other (specify)

5) How important do you think is it for all people to know how to perform CPR?

Not important very important

6) How likely are you to read or look up for additional material regarding CPR, after this session?

Not likely extremely likely

7) How likely are you to encourage other people to learn CPR?

Not likely extremely likely

8) When do you think is the ideal time for you to learn CPR

a) Now is the correct time

b) Should have got the opportunity to learn earlier

c) Should be learnt later

9) How likely are you to attend a certified BLS training session at your own expense?

Not likely Very likely

10) How likely are you to volunteer to train others to do CPR?

Not likely Very likely

11) After training, which components are you willing/comfortable to perform if you encounter a victim who needs CPR (you can tick one or more options)

1. Assessment of the victim, calling for help
2. Giving chest compressions
3. Giving breaths
4. Using AED

12) Which components do you think are you likely to perform correctly on your own in adult CPR after this training? (You can tick one or more option)

1. Assessment of the victim, calling for help
2. Giving chest compressions
3. Giving breaths
4. Using AED

**Additional questions asked only for the intervention group**

1. Did the talk by the medical student about experience with CPR, influence your attitude towards CPR?

Not likely (0) very likely (10)

1. Did the talk by the medical student about experience with CPR, motivate you to learn CPR?

Not likely (0) very likely (10)

1. If you found the talk by the medical student about experience with CPR useful, can you specify in what way it was useful? (Open-ended)
