# Supplementary material for: Influence of pretesting and a near peer sharing real life experiences on CPR training outcomes in first year medical students: a non-randomized quasi-experimental study
Source: BMC Med Educ. 2022 Jun 6;22:434. doi: 10.1186/s12909-022-03506-4 (PMC9172151; doi:10.1186/s12909-022-03506-4)
Supplement: Supplementary file 3 — Additional file 3. [file 12909_2022_3506_MOESM3_ESM.docx]

| **Skill evaluation checklist** | | | |
| --- | --- | --- | --- |
|  | Not performed  0 | Performed but partially correct  1 | Correctly performed  2 |
| Checks for responsiveness |  |  |  |
| Activates emergency response system |  |  |  |
| **Assessment of pulse and breathing** | | | |
| Checks pulse at the correct Location |  |  |  |
| Checks for breathing |  |  |  |
| Assesses for at least 5s but not more than 10 s |  |  |  |
| **Chest compressions** | | | |
| Correct location of compression |  |  |  |
| Correct depth of compression |  |  |  |
| Correct technique of compressions  (uses two hands , shoulders over sternum , no bending of elbows ) |  |  |  |
| 30 compressions given between 15 -18 s |  |  |  |
| **Breaths** |  |  |  |
| Correct technique of head tilt, chin lift |  |  |  |
| Delivers each breath over 1 s avoiding overventilation |  |  |  |
| Breaths just enough to cause visible chest rise |  |  |  |
| Takes less than 10 seconds to give 2 breaths |  |  |  |
| Correct use of bag mask device in 2 rescuer CPR |  |  |  |
| **Operation of AED device** | | | |
| Switches on AED |  |  |  |
| Attaches pads at the correct location and connected the wires to AED |  |  |  |
| CPR stopped during rhythm analysis by AED |  |  |  |
| Switches roles |  |  |  |
| Clears the victim before giving shock |  |  |  |
| Presses the button, then resumes CPR without switching off the device |  |  |  |
| TOTAL SCORE |  | | |
