# Supplementary material for: Influence of pretesting and a near peer sharing real life experiences on CPR training outcomes in first year medical students: a non-randomized quasi-experimental study
Source: BMC Med Educ. 2022 Jun 6;22:434. doi: 10.1186/s12909-022-03506-4 (PMC9172151; doi:10.1186/s12909-022-03506-4)
Supplement: Supplementary file 4 — Additional file 4. [file 12909_2022_3506_MOESM4_ESM.docx]

**Thematic Analysis of the Student Feedback**

Table 1: A detailed thematic analysis of the students’ responses for the usefulness of peer sharing real-life experience during the BLS training

| Responses | Codes | Themes |
| --- | --- | --- |
| It motivates a person to learn this personal development skill | Motivates | Learning motivation/Inspiration |
| Highly motivational and being an MBBS student it's absolutely necessary to learn CPR | Need to learn |  |
| A real life scenario coming from someone in whose place one would be 2yrs from now makes it seem very important and influences you. | Influence |  |
| It showed that CPR could be needed anywhere, anytime, so we need to learn that | Need to learn |  |
| It showed how real this thing was and it can really save lives if done at the correct time and done in a correct manner. It also motivated me to actually learn CPR and not just treat it like the other Foundation Course class. | Learning motivation |  |
| It was good to know how CPR is such an important skill to learn, whether you are a medical student or not. It can be very useful anytime and anywhere and can save a life. | Usefulness |  |
| It was very inspirational. | Inspirational |  |
| The talk by the medical student was very inspiring and highlighted the importance of knowing correct technique of CPR. | Inspirational |  |
| It motivated me to do it with self confidence | Self confidence |  |
| Removal of fear and helped me attain self confidence |  |  |
| She talked about her own experience so now I am more confident to apply this knowledge practically. |  |  |
| Learnt a lifesaving technique. Now I have the confidence to raise my hand if required doing CPR in any situation. |  |  |
| It was inspiring to listen to a medical student about saving life using CPR | Help/Encourage others | Helping/Saving lives |
| I learnt to perform CPR and will also encourage others to learn | Encourage others |  |
| As the student told, we can get caught in any kind of emergency and it might occur anywhere, so we can actually save a life by learning this, no medical knowledge is required just a simple procedure | Saving a life |  |
| It may give me an opportunity to learn to save somebody in the future |  |  |
| It was truly inspiring and after listening to it, I would highly recommend everyone to learn CPR |  |  |
| It encouraged me to know more about this |  |  |
| She was quite motivating and at the same time realistic in her approach towards explaining. |  |  |
| It was motivating, inspiring and taught me to believe in my abilities and skills. |  |  |
| It was inspiring. Shows how CPR could save one's life. |  |  |
| It inspired all of us to know that now we are in a position to save lives |  |  |
| It gave me the confidence to provide help to the needed, without any hesitation. | Helping in emergencies |  |
| Motivate us to help our fellow humans whenever they are in need |  |  |
| It really encouraged me to learn CPR as it will be very helpful to save anyone in need |  |  |
| Helping anyone in an emergency |  |  |
| We must help a person in need in the best possible way we can. |  |  |
| Can help the needy |  |  |
| The talk inspired me to learn CPR and not be afraid to lend a hand to those in need. |  |  |
| It was inspiring and genuine |  |  |
| Helped me change my attitude towards CPR | Realization of a situation | Change in attitude |
| It inspired us to learn CPR and her experience made us realise the real importance of learning CPR |  |  |
| Made me realize the importance of knowing CPR, and also that u can’t save everyone |  |  |
| Made me understand the importance of learning CPR and I realised how anything can happen at any point of time |  |  |
| She made us realise that as a medical student or a normal human being it is very important to step into a medical emergency and the patient cannot be saved all the time |  |  |
| It was a real experience and not a hypothetical scenario. It helped me realise the real importance of having the power to save a life |  |  |
| It was good in that it made me aware of a real life situation | Real-life scenario  Application-based | Awareness |
| Real life scenarios always get us back to reality of the need to learn medical techniques | Relevance |  |
| Was useful as I was not aware of how important CPR is. | Importance of CPR |  |
| I did not know how to perform CPR before this session and how important it is. Now I will be able to save a person’s life |  |  |
| It gave me an idea about how clueless the society is about CPR and most of the people do not have the correct training and how all this effects a person's life in emergency. |  |  |
| It made me realise the importance of BLS training. | Importance of learning |  |
| I realised that even though I am not a trained medical professional, there is still a chance that I can save someone's life and learning CPR is what I needed to do. | Saving life |  |
| It was very realistic as the possibility of positive or negative results after CPR was told to us |  |  |
| It has enlightened me to the various procedures that are worth understanding to help me give my best in saving someone's life in case of need. |  |  |
| Realized the importance of learning a technique that could save a person’s life |  |  |
| Made me realize that emergencies do happen in real life and it can happen around me anytime anywhere. | Preparedness |  |
| The real life experiences conveyed how a simple skill can save the life of a person. Various techniques to save the lives of adults and infants were demonstrated to prepare us for handling emergency situations which we as future doctors must know |  |  |
| Inspired me to be brave in times of stress |  |  |
| It made me realise the importance of today’s session beforehand and how it could actually impact some situation later in life. In addition, knowing that emergencies can happen, prepared me to try my best in case I have to administer CPR. |  |  |
| It was useful, as I understood that what we are learning here could be applicable anywhere. I realized that at times we may not be able to perceive this. We should always be ready. | Readiness |  |
| Informative | Information | Knowledge gain |
| It enhanced my knowledge |  |  |
| It was informative and the fact that we got to perform it (on mannequins) helped me gain a better understanding | Better understanding |  |
